# The Natural Pigment Violacein Potentially Suppresses the Proliferation and Stemness of Hepatocellular Carcinoma Cells In Vitro

**DOI:** 10.3390/ijms221910731

**Published:** 2021-10-03

**Authors:** Yu Jin Kim, Nayeong Yuk, Hee Jeong Shin, Hye Jin Jung

**Affiliations:** 1 Department of Life Science and Biochemical Engineering, Sun Moon University, Asan 31460, Korea; petaldew17@naver.com (Y.J.K.); gmlwjd903@naver.com (H.J.S.); 2 Department of Pharmaceutical Engineering and Biotechnology, Sun Moon University, Asan 31460, Korea; nayeong7249@naver.com; 3 Genome-Based BioIT Convergence Institute, Sun Moon University, Asan 31460, Korea

**Keywords:** violacein, hepatocellular carcinoma, proliferation, apoptosis, stemness

## Abstract

Hepatocellular carcinoma (HCC) is a malignant type of primary liver cancer with high incidence and mortality, worldwide. A major challenge in the treatment of HCC is chemotherapeutic resistance. It is therefore necessary to develop novel anticancer drugs for suppressing the growth of HCC cells and overcoming drug resistance for improving the treatment of HCC. Violacein is a deep violet-colored indole derivative that is produced by several bacterial strains, including *Chromobacterium violaceum*, and it possesses numerous pharmacological properties, including antitumor activity. However, the therapeutic effects of violacein and the mechanism underlying its antitumor effect against HCC remain to be elucidated. This study is the first to demonstrate that violacein inhibits the proliferation and stemness of Huh7 and Hep3B HCC cells. The antiproliferative effect of violacein was attributed to cell cycle arrest at the sub-G1 phase and the induction of apoptotic cell death. Violacein induced nuclear condensation, dissipated mitochondrial membrane potential (MMP), increased generation of reactive oxygen species (ROS), activated the caspase cascade, and upregulated p53 and p21. The anticancer effect of violacein on HCC cells was also associated with the downregulation of protein kinase B (AKT) and extracellular signal-regulated kinase (ERK)1/2 signaling. Violacein not only suppressed the proliferation and formation of tumorspheres of Huh7 and Hep3B cancer stem-like cells but also reduced the expression of key markers of cancer stemness, including CD133, Sox2, Oct4, and Nanog, by inhibiting the signal transducer and activator of transcription 3 (STAT3)/AKT/ERK pathways. These results suggest the therapeutic potential of violacein in effectively suppressing HCC by targeting the proliferation and stemness of HCC cells.

## 1. Introduction

Hepatocellular carcinoma (HCC) is a primary malignancy of the liver, and it accounts for approximately 75% of all cases of liver cancer [1]. In 2018, HCC was the seventh most prevalent cancer worldwide and the second most common cause of cancer-related mortality [2]. In particular, the incidence of HCC is high in Asia and Africa [3]. The primary causes of HCC include chronic hepatitis B virus (HBV) or chronic hepatitis C virus (HCV) infections, alcoholic cirrhosis, and non-alcoholic steatohepatitis (NASH) [4]. To date, the early diagnosis and effective treatment of HCC continues to be a challenge. Most patients are asymptomatic and develop symptoms at an advanced stage of the disease. The long-term prognosis of treatment is good when detected very early; however, for the majority of patients with HCC, the disease is detected at a stage where surgical treatment is no longer feasible [5]. Therefore, most patients with HCC require chemotherapy, which involves the use of chemical agents for destroying cancer cells and inhibiting the growth of new cancer cells [6]. Chemotherapy, along with radiation therapy, surgery, and immunotherapy, have been used for the treatment of HCC for many years, but the progress is poor due to the high recurrence rate and frequent cirrhosis [7]. Sorafenib, a tyrosine kinase inhibitor, inhibits the activities of Raf kinase and vascular endothelial growth factor receptor (VEGFR), and it is the most commonly used targeted chemotherapy drug for the treatment of HCC. However, it has been demonstrated that sorafenib improves patient survival by only about 7–10 months [8,9]. Other kinase inhibitors that have been recently approved for the treatment of HCC include regorafenib and lenvatinib. However, the treatment benefits of these drugs have not been shown to be significantly superior to those of sorafenib [10,11]. Therefore, for the effective treatment of HCC, continued studies are necessary for identifying novel candidate drugs to overcome drug resistance.

Apoptosis, a genetically encoded cell death program, is associated with characteristic morphological and biochemical changes, including chromatin condensation, DNA fragmentation, and loss of mitochondrial membrane potential (MMP) [12,13]. Apoptosis is activated by intrinsic and extrinsic pathways and is modulated by a multitude of factors, including intracellular mediators of signal transduction and nuclear proteins that regulate gene expression, DNA replication, and the cell cycle [13]. Representatively, the activation of caspases, the increased expression of tumor suppressor p53, and the generation of reactive oxygen species (ROS) are major factors for the initiation of apoptosis and are molecular targets of numerous anticancer drugs [14,15]. As the main goal of cancer therapy is to eliminate cancer cells while minimizing damage to normal cells, the specific apoptosis of cancer cells can serve as a promising therapeutic strategy against cancer [16]. Therefore, drugs that induce the specific apoptosis of HCC cells can be considered as a potential treatment option for the disease.

Cancer stem cells (CSCs), also known as tumor-initiating cells (TICs), have stem cell-like properties and are defined as a small subpopulation of cancer cells that contributes to tumor diversity and heterogeneity [17,18]. CSCs are more tumorigenic and resistant to anticancer therapies than non-stem cancer cells [18,19]. Therefore, CSCs are closely related to tumor carcinogenesis, recurrence, and metastasis [18,19]. Mature hepatocytes, hepatoblasts, and bile cells can transform into liver CSCs (LCSCs) during liver injury and trigger hepatic regeneration or lead to a state of oncogenic dedifferentiation [20]. LCSCs are being increasingly recognized as responsible for the initiation, relapse, metastasis, and chemoresistance of HCC [21]. Therefore, the suppression of LCSCs is a promising strategy for the effective treatment of HCC and the prevention of relapse.

**Figure 1:**
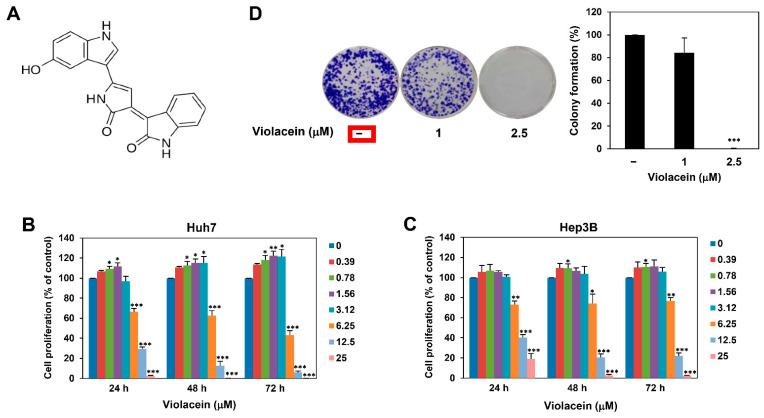
Violacein inhibits the proliferation of Huh7 HCC cells. (**A**) Chemical structure of violacein. (**B**,**C**) Effect of violacein on the proliferation of Huh7 and Hep3B cells. The cells were treated with violacein at various concentrations (0–25 µM) for 24, 48, or 72 h. Cell proliferation was measured using the CellTiter-Glo^®^ luminescent assay system. (**D**) Effect of violacein on the colony-forming ability of Huh7 cells. The cells were incubated in the absence or presence of violacein (1 and 2.5 µM) for 12 days. The cell colonies were detected by crystal violet staining. * *p* < 0.05, ** *p* < 0.01, *** *p* < 0.001 vs. the control.

Natural products are a highly useful source of bioactive molecules and serve as an important and valuable resource for drug development [22]. Several preclinical and research findings have demonstrated that bioactive compounds derived from natural products show considerable potential in the prevention and treatment of several types of cancer [22,23]. Violacein, an indole derivative produced as a secondary metabolite of *Chromobacterium violaceum* (Figure 1A), is a purple-colored natural pigment that exhibits various biological properties, including antimicrobial, antiparasitic, anti-inflammatory, and antitumor activities [24]. Previous studies have demonstrated that violacein exhibits anticancer activity by inducing apoptosis in various types of cancer, including breast cancer, colon cancer, lung cancer, and leukemia [25,26,27]. However, the mechanism underlying the anticancer effect of violacein in HCC remains to be elucidated.

In this study, the anticancer effects and molecular mechanism underlying the anticancer property of violacein in Huh7 and Hep3B HCC cells were investigated. The results of our study demonstrate that violacein effectively inhibited the proliferation of HCC cells by upregulating the apoptotic pathways and also eradicated the stem-like features by downregulating major cancer stemness regulators in HCC cells. We therefore suggest that the natural compound violacein can serve as a potential therapeutic alternative for HCC.

## 2. Results

### 2.1. Violacein Inhibits the Proliferation of Huh7 HCC Cells

In order to examine whether violacein affects the proliferation of HCC cells, Huh7 cells were treated with violacein at various concentrations (0–25 μM) for 24, 48, or 72 h. Cell proliferation was subsequently measured using the ATP-monitoring luminescence assay. As depicted in Figure 1B, treatment with violacein showed a biphasic dose–response on the proliferation of Huh7 cells. At low concentrations (0.39–3.12 μM), violacein induced the proliferation of Huh7 cells. However, at higher concentrations (6.25–25 μM), it inhibited the Huh7 cell proliferation, with IC_50_ values of 7.97, 6.71, and 6.10 μM at 24, 48, and 72 h, respectively. A similar pattern of dose–response was observed in different HCC cells, Hep3B (Figure 1C). The IC_50_ values of violacein for Hep3B cells were determined to be 8.01, 8.41, and 8.23 μM at 24, 48, and 72 h, respectively. These data indicate that violacein may have a biphasic effect on the proliferation of HCC cells by modulating the action of molecular targets involved in HCC cell proliferation in a concentration-dependent biphasic manner.

We next evaluated the effect of violacein on the formation of Huh7 cell colonies. Colony formation was observed on day 12 after treatment with violacein. As depicted in Figure 1D and Figure S1, 2.5 μM violacein completely suppressed the colony-forming ability of Huh7 cells. Taken together, these results demonstrate that violacein has inhibitory potential on the proliferation of HCC cells.

### 2.2. Violacein Promotes Apoptotic Characteristics in Huh7 HCC Cells

**Figure 2:**
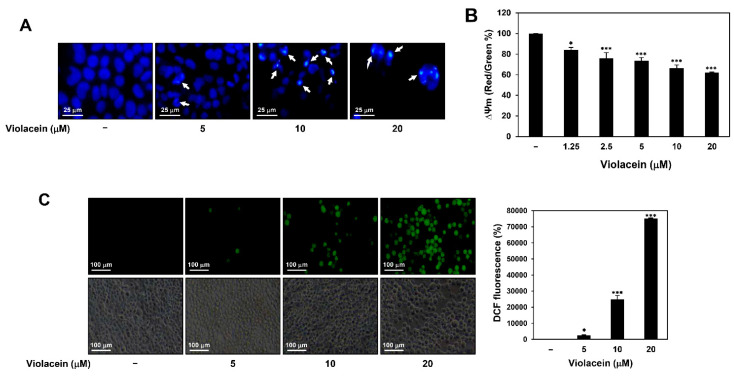
Violacein promotes apoptotic characteristics in Huh7 HCC cells. (**A**) Effect of violacein on the nuclear morphology. Huh7 cells were treated with violacein (5, 10, and 20 µM) for 24 h. Changes in nuclear morphology were monitored by DAPI staining under a fluorescence microscope. The condensed nuclei are indicated by white arrows. (**B**) Effect of violacein on the MMP. Huh7 cells were treated with violacein (0–20 µM) for 24 h and stained with JC-1. The fluorescence intensity of J-aggregates and JC-1 monomers was detected by a multimode microplate reader. (**C**) Effect of violacein on the generation of intracellular ROS. Huh7 cells were treated with violacein (5, 10, and 20 µM) for 6 h. The levels of ROS were detected with DCFH-DA using a fluorescence microscope and were further quantified by densitometry. * *p* < 0.05, *** *p* < 0.001 vs. the control.

In order to further investigate the mechanisms underlying the antiproliferative effect of violacein in Huh7 HCC cells, we determined by 4′,6-diamidino-2-phenylindole (DAPI) staining whether violacein causes nuclear morphological changes in Huh7 cells. As depicted in Figure 2A, treatment with violacein caused nuclear condensation, a prominent hallmark of apoptosis.

The loss of MMP (Δψm) can induce early apoptosis [12,13]. We therefore evaluated the effect of treatment with violacein on the MMP in Huh7 cells using the cationic 5,5′,6,6′-tetra-chloro-1,1′,3,3′-tetraethylbenzimidazol-carbocyanine iodide (JC-1) fluorescent dye. As depicted in Figure 2B, violacein significantly decreased the ratio of intensity of red/green fluorescence in a dose-dependent manner, indicating that violacein caused the loss of MMP in Huh7 cells.

Intracellular ROS play a central role in biological processes, especially in the induction of apoptosis [12,15]. In order to assess whether violacein affects the generation of ROS in Huh7 cells, the level of intracellular ROS was measured using 2′,7′-dichlorofluorescein diacetate (DCFH-DA). Violacein significantly increased the accumulation of ROS in a dose-dependent manner (Figure 2C). Taken together, these results suggest that violacein may inhibit the proliferation of Huh7 HCC cells by inducing apoptotic cell death.

### 2.3. Violacein Induces Cell Cycle Arrest at the Sub-G1 Phase and Cellular Apoptosis in Huh7 HCC Cells

**Figure 3:**
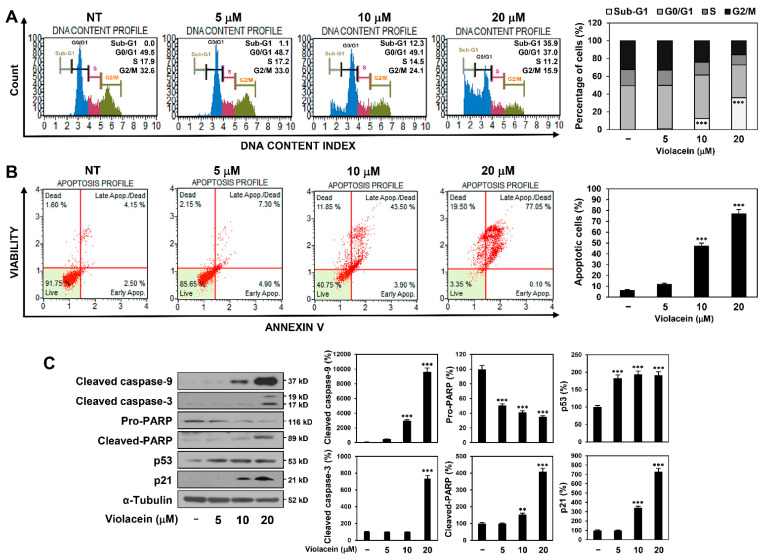
Violacein induces cell cycle arrest at the sub-G1 phase and cellular apoptosis in Huh7 HCC cells. (**A**–**C**) Huh7 cells were treated with violacein (5, 10, and 20 µM) for 24 h. (**A**) Effect of violacein on the cell cycle. The cell cycle distribution was evaluated using a Muse Cell Analyzer with Muse^®^ Cell Cycle kit. (**B**) Effect of violacein on the apoptotic cell death. Apoptotic cells were detected using a Muse Cell Analyzer with Muse^®^ Annexin V & Dead Cell kit. (**C**) Effect of violacein on the expression of apoptosis regulators. Protein levels were detected by Western blot analysis using specific antibodies and were further quantified by densitometry. α–tubulin levels were used as an internal control. ** *p* < 0.01, *** *p* < 0.001 vs. the control.

In order to further explore the role of apoptosis in the inhibitory effect of violacein on the proliferation of HCC cells, we investigated the effect of violacein on the progression of the cell cycle in Huh7 cells by flow cytometric analysis. DNA fragmentation can be measured by flow cytometry using the sub-G1 assay. The small DNA fragments generated during apoptosis leak out of cells, decreasing the total DNA content of apoptotic cells. By staining DNA with PI, hypodiploid apoptotic cells can be detected as a “sub-G1” population of the PI histogram. As depicted in Figure 3A, treatment with violacein significantly increased the population of cells in the sub-G1 phase and decreased the population of cells in the G0/G1, S, and G2/M phases, compared with those of the untreated control group. These data imply that violacein caused nuclear fragmentation in the HCC cells and consequently arrested cell cycle progression at the sub-G1 phase, which represents apoptotic cells.

Next, cellular apoptosis was analyzed by flow cytometry by Annexin V-FITC and PI dual labeling. Treatment with violacein significantly increased the proportion of apoptotic cells in a dose-dependent manner compared with that in the untreated control group (Figure 3B). These data demonstrate that violacein induced apoptosis in Huh7 HCC cells.

In order to further elucidate the molecular mechanism underlying violacein-induced apoptosis, we assessed the effect of violacein on the expression of crucial mediators of apoptosis in Huh7 cells. Caspases play an important role in apoptosis and are activated by proteolytic cleavage [12,13]. Upon induction of apoptosis, cytochrome c released from mitochondria associates with caspase-9 and apoptotic protease activating factor-1 (Apaf-1). The complex processes caspase-9 (47 kD) into several subunits, including a p37 fragment (37 kD). The cleaved caspase-9 further processes caspase-3 (35 kD) that is a critical executioner of apoptosis. The cleaved caspase-3 (17/19 kD) is responsible for the proteolytic cleavage (89 kD) of downstream substrates involved in apoptotic changes, including a 116 kD nuclear poly (ADP-ribose) polymerase (PARP). As depicted in Figure 3C, treatment with violacein increased the levels of cleaved caspase-9, cleaved caspase-3, and cleaved PARP. Additionally, violacein upregulated the expression of the tumor suppressor p53 and its transcriptional target, p21, which are implicated in both cell cycle arrest and apoptosis. These results suggest that violacein may induce cellular apoptosis via the activation of p53- and caspase-mediated apoptotic pathways in Huh7 HCC cells.

### 2.4. Violacein Downregulates Protein Kinase B (AKT) and Extracellular Signal-Regulated Kinase (ERK) Signaling in Huh7 HCC Cells

**Figure 4:**
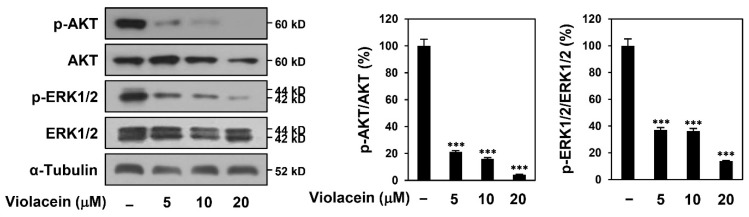
Violacein downregulates AKT and ERK signaling in Huh7 HCC cells. The cells were treated with violacein (5, 10, and 20 µM) for 1 h. Protein levels were detected by Western blot analysis using specific antibodies and were further quantified by densitometry. α–tubulin levels were used as an internal control. *** *p* < 0.001 vs. the control.

Aberrantly activated AKT and ERK signaling contributes to the survival and proliferation of several types of cancers, including HCC [28]. AKT is a serine/threonine kinase that promotes cell survival by inhibiting apoptosis. ERK1 and ERK2 isoforms are 44 and 42 kD serine/threonine kinases, respectively, belonging to the mitogen-activated protein kinase family. We therefore examined whether violacein affects the key signaling pathways in Huh7 cells. In particular, to clarify whether the inhibitory effect of violacein on HCC cell proliferation is due to inactivation of the signaling pathways, the effect of the compound was evaluated at the early time point of 1 h. As depicted in Figure 4, violacein more effectively inhibited the expression levels of phosphorylated forms compared with unphosphorylated AKT and ERK1/2 proteins. These data indicate that the antiproliferative effect of violacein on Huh7 cells could be mediated via the downregulation of AKT and ERK signaling pathways.

### 2.5. Violacein Inhibits the Proliferation and Formation of Tumorspheres of Huh7 Cancer Stem-Like Cells

Accumulating evidence has revealed that CSCs, a subpopulation of tumor cells, facilitate metastasis, recurrence, and resistance to chemotherapy or radiotherapy in HCC [17]. We therefore further investigated the effect of violacein on the stemness of Huh7 cells. In order to propagate the cancer stem-like cells, Huh7 cells were grown in serum-free spheroid suspension culture [29]. The 3D spheroid cell culture is known to stimulate in vivo cellular conditions better in comparison with 2D cell culture, and it is widely used to increase a subpopulation of cancer cells with stem cell-like properties, thereby providing new insights into cancer treatment and CSC research [29]. As depicted in Figure S2, the expression levels of several key stemness-related markers were remarkably increased in the Huh7 tumorsphere cells cultured in serum-free media containing EGF and bFGF compared to the Huh7 adherent cells cultured in 10% FBS-supplemented media. These data indicate that the serum-free spheroid culture can expand the CSC population from HCC cell lines.

**Figure 5:**
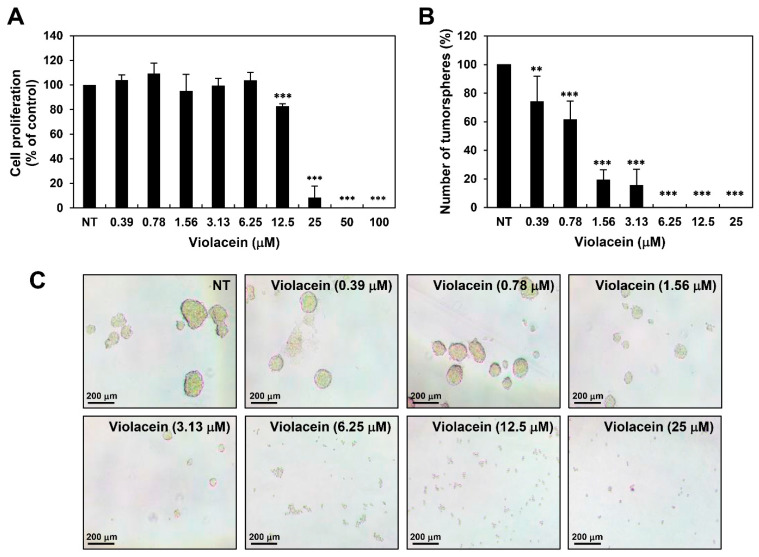
Violacein inhibits the proliferation and formation of tumorspheres of Huh7 cancer stem-like cells. (**A**) Effect of violacein on the proliferation of Huh7 cancer stem-like cells. The cells were treated with violacein at various concentrations (0–100 µM) and were incubated with the CSC culture media for 7 days. Cell proliferation was measured using the CellTiter-Glo^®^ luminescent assay system. (**B**,**C**) Effect of violacein on the tumorsphere-forming ability of Huh7 cancer stem-like cells. The cells were treated with violacein at various concentrations (0–25 µM) and were incubated with the CSC culture media for 7 days. The number of tumorspheres in each well was counted under an optical microscope. ** *p* < 0.01, *** *p* < 0.001 vs. the control.

The Huh7 cancer stem-like cells were treated with violacein at various concentrations (0–100 μM) for 7 days, and cell proliferation was measured using the ATP-monitoring luminescence assay. As depicted in Figure 5A, treatment with violacein inhibited the proliferation of Huh7 cancer stem-like cells, and the IC_50_ value was determined to be 16.47 μM. Furthermore, both the size and number of tumorspheres formed by the Huh7 cancer stem-like cells were effectively reduced following treatment with violacein (Figure 5B,C). In particular, the tumorsphere-forming ability was remarkably suppressed by violacein, even at concentrations 10 times lower than the IC_50_ value for inhibiting the proliferation of Huh7 cancer stem-like cells. We further identified that violacein potently inhibited the tumorsphere-forming ability of Hep3B cancer stem-like cells (Figure S3). These data suggest the possible therapeutic potential of violacein in eradicating CSCs in HCC.

### 2.6. Violacein Downregulates Cancer Stemness-Related Markers and Signal Transducer and Activator of Transcription 3 (STAT3)/AKT/ERK Signaling in Huh7 Cancer Stem-Like Cells

**Figure 6:**
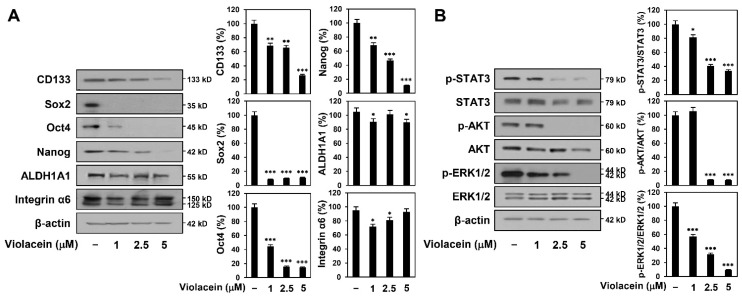
Violacein downregulates cancer stemness-related markers and STAT3/AKT/ERK signaling in Huh7 cancer stem-like cells. (**A**,**B**) The cells were treated with violacein (1, 2.5, and 5 µM) and were incubated with the CSC culture media for 72 h. Protein levels were detected by Western blot analysis using specific antibodies and were further quantified by densitometry. β-actin levels were used as an internal control. * *p* < 0.05, ** *p* < 0.01, *** *p* < 0.001 vs. the control.

We next evaluated whether violacein regulates the expression of key stemness-related markers in the CSCs in HCC. The results demonstrate that violacein markedly suppressed the expression of CD133, Sox2, Oct4, and Nanog in Huh7 cancer stem-like cells (Figure 6A). However, it slightly inhibited the expression of aldehyde dehydrogenase 1A1 (ALDH1A1) at concentrations of 1 and 5 μM, while it did not reduce the protein levels at 2.5 μM. Meanwhile, violacein suppressed the expression of integrin α6 (125 kD reduced and 150 kD nonreduced forms) at 1 and 2.5 μM treatment, but not at 5 μM.

It has been reported that STAT3 signaling regulates the expression of several CSC markers, including CD133 and Nanog, in HCC [30]. The AKT and ERK signaling pathways are also involved in the maintenance of self-renewal in CSCs in HCC [31,32]. Treatment with violacein inhibited the phosphorylation of STAT3, AKT, and ERK1/2 without affecting the total protein expression (Figure 6B). These results demonstrate that violacein may inhibit the stem-like features of Huh7 HCC cells by inactivating the STAT3/AKT/ERK pathway.

## 3. Discussion

HCC is the most common primary malignancy of the liver, with a high mortality rate that is attributed to difficulties in early diagnosis, high recurrence rate, and frequent cirrhosis [1,7]. Therefore, the continued exploration and development of novel anticancer agents is necessary for reducing the rate of recurrence of HCC and achieving effective chemotherapy.

Violacein is a natural pigment with numerous biological activities, and it is obtained from several bacterial strains, including *C. violaceum* [24]. Several studies have demonstrated that violacein can induce apoptosis in a variety of cancer cells, including breast cancer, colon cancer, lung cancer, and leukemia [25,26,27]. It has been demonstrated that violacein induces the accumulation of ROS in tumors by increasing the expression of genes related to oxidative stress, including GSK3β and TNF-α, and induces apoptosis by upregulating the expression of apoptosis-inducing genes, including p53, caspase-3, caspase-8, caspase-9, and PARP [25,26]. However, the anticancer activity and mechanism underlying the antitumor effect of violacein in HCC are yet to be investigated. This study was the first to evaluate the anticancer effect of violacein in Huh7 and Hep3B HCC cells.

The evasion of apoptosis is the hallmark of cancer and results in cancer progression and drug resistance [33]. Therefore, the induction of apoptosis through the reactivation of major apoptotic pathways and the inhibition of cell survival signaling cascades can offer a potential strategy for efficiently suppressing the growth of HCC cells. The results of our study demonstrate that violacein effectively inhibited the proliferation of HCC cells by inducing apoptosis. Violacein causes cell cycle arrest at the sub-G1 phase and activates the key regulatory mechanisms of apoptosis, including nuclear condensation, dissipation of MMP, increased generation of ROS, activation of the caspase cascade, and upregulation of p53 and p21. Additionally, the antiproliferative effect of violacein on HCC cells was mediated via the downregulation of AKT and ERK1/2 signaling pathways, which play an important role in sustaining proliferation and preventing the apoptosis of tumor cells [28]. These findings demonstrate that violacein potently inhibits the proliferation of HCC cells via activation of apoptotic pathways and deactivation of survival pathways.

CSCs are a small subpopulation of cancer cells with stem cell properties, including self-renewal and multilineage differentiation, and they have been identified in many tumors, including liver cancer [17,18,19]. It has been demonstrated that LCSCs, also known as hepatic cancer stem cells, contribute to the initiation, relapse, metastasis, and chemoresistance of HCC [20,21]. Therefore, LCSCs are a vital target for the successful treatment of HCC. Notably, the results of our study demonstrate that violacein significantly inhibited the proliferation and formation of tumorspheres of Huh7 and Hep3B cancer stem-like cells. Additionally, violacein effectively suppressed the key stemness-related markers and signaling pathways in cells.

Accumulating evidence has revealed that several cell surface markers, transcription factors, and molecular signaling pathways are activated in LCSCs, which mediate the development and maintenance of LCSCs [34,35]. The CD133 cell surface marker has been detected in numerous types of CSCs, and CD133-positive HCC has stronger carcinogenicity and proliferative capacity [34]. The key regulatory transcription factors, including Sox2, Oct4, and Nanog, not only play a critical role in the maintenance and self-renewal of LCSCs but also contribute to the malignancy of HCC via mechanisms related to drug resistance, including epithelial–mesenchymal transition (EMT) [21,34,35,36]. ALDH1A1, an isoform of the aldehyde dehydrogenase enzyme, partakes in the clearance of toxic aldehydes and reduces oxidative stress, which consequently leads to the survival of CSCs and poor prognosis [37]. Integrin α6, the receptor for the extracellular matrix (ECM) protein laminin, plays an important role in maintaining the CSC niche and regulating homeostasis in CSCs [36]. In this study, violacein noticeably reduced the expression levels of CD133, Sox2, Oct4, and Nanog in Huh7 cancer stem-like cells. However, it differently regulated the expression levels of ALDH1A1 and integrin α6 at each treatment concentration.

Additionally, several molecular signaling pathways, including the STAT3, AKT, and ERK pathways, are aberrantly activated in HCC, leading to the dysregulation of downstream target genes that control proliferation, survival, invasion, and stemness [30,31,32]. Activated STAT3 translocates to the nucleus and induces the transcription of several markers of CSCs in HCC, including CD133 and Nanog, thereby promoting the maintenance and self-renewal of LCSCs [38,39]. The AKT and ERK signaling pathways are also implicated in the proliferation, metabolism, and differentiation of LCSCs via upregulation of the expression of Sox2, Oct4, and Nanog [31,32,40]. The results of our study demonstrate that violacein significantly inhibited the phosphorylation of STAT3, AKT, and ERK1/2 without reducing the total protein levels in Huh7 cancer stem-like cells. These findings suggest that violacein may suppress the cancer stem-like features of HCC cells by inhibiting the expression of key cancer stemness markers, including CD133, Sox2, Oct4, and Nanog, via downregulation of the STAT3/AKT/ERK signaling pathways.

Taken together, the study demonstrates the therapeutic potential of violacein in effectively suppressing HCC by targeting both the proliferation and stemness of HCC cells. However, further in vivo experiments with animal models and identification of the primary molecular target of violacein are necessary before violacein can be used for the clinical treatment of HCC.

## 4. Materials and Methods

### 4.1. Materials

Violacein was kindly provided by Prof. Jae Kyung Sohng (Sun Moon University, Asan, Korea) and dissolved in dimethyl sulfoxide (DMSO) at a concentration of 100 mM. Dulbecco’s modified Eagle’s medium (DMEM) and Roswell Park Memorial institute-1640 (RPMI-1640) were purchased from Corning Cellgro (Manassas, VA, USA). DMEM/F12 and trypsin were purchased from HyClone (Marlborough, MA, USA). Epidermal growth factor (EGF) and basic fibroblast growth factor (bFGF) were obtained from Prospecbio (East Brunswick, NJ, USA). Fetal bovine serum (FBS), B-27 serum-free supplement, L-glutamine, and penicillin/streptomycin were obtained from Gibco (Grand Island, NY, USA). Penicillin-streptomycin-amphotericin B and Accutase were obtained from Lonza (Walkersville, MD, USA) and EMD Millipore (Temecula, CA, USA), respectively. Heparin, DAPI, DCFH-DA, and JC-1 were purchased from Sigma-Aldrich (St. Louis, MO, USA). The CellTiter-Glo^®^ 2.0 Cell Viability Assay kit was purchased from Promega (Madison, WI, USA). The Muse^®^ Annexin V & Dead Cell and Muse^®^ Cell Cycle kits were purchased from Luminex (Austin, TX, USA). The antibodies against cleaved caspase-9 (cat. no. 9501), cleaved caspase-3 (cat. no. 9661), PARP (cat. no. 9542), p53 (cat. no. 2524), p21 (cat. no. 2947), ALDH1A1 (cat. no. 12035), Integrin α6 (cat. no. 3750), Sox2 (cat. no. 3579), Oct4 (cat. no. 2750), Nanog (cat. no. 3580), CD133 (cat. no. 64326), phospho-STAT3 (cat. no. 9145), STAT3 (cat. no. 9139), phospho-AKT (cat. no. 4060), AKT (cat. no. 9272), phospho-ERK1/2 (cat. no. 9101), ERK1/2 (cat. no. 9102), α-tubulin (cat. no. 2144), rabbit IgG (cat. no. 7074), and mouse IgG (cat. no. 7076) were purchased from Cell Signaling Technology (Danvers, MA, USA). Anti-β-actin antibody (cat. no. ab6276) was purchased from Abcam (Cambridge, UK).

### 4.2. Cell Culture

Huh7 and Hep3B human HCC cells were purchased from the Korean Cell Line Bank (Seoul, Korea). The Huh7 and Hep3B cells were grown in DMEM supplemented with 10% FBS and 1% penicillin–streptomycin–amphotericin B, respectively. The Huh7 and Hep3B cancer stem-like cells were cultured in DMEM/F12 containing 1 × B-27, 5 µg/mL heparin, 2 mM L-glutamine, 20 ng/mL EGF, 20 ng/mL bFGF, and 1% penicillin/streptomycin. All the cells were maintained at 37 °C in a humidified CO_2_ incubator with 5% CO_2_ (Thermo Scientific, Vantaa, Finland).

### 4.3. Cell Proliferation Assay

Cell proliferation was quantitatively evaluated using the CellTiter-Glo^®^ 2.0 Cell Viability Assay kit. Briefly, the cells (5 × 10^3^ cells/well) were seeded in a 96-white-well culture plate and treated with violacein at various concentrations. Following incubation for the indicated durations, 20 μL of substrate solution was added to each well, and the culture plate was shaken for 2 min and subsequently incubated for 8 min in the dark. Luminescence was detected using a multimode microplate reader (BioTek, Inc., Winooski, VT, USA). The IC_50_ values determined from the obtained data were analyzed by the curve-fitting program in GraphPad Prism, version 5 (GraphPad Software, La Jolla, CA, USA).

### 4.4. Colony Formation Assay

Huh7 cells (1 × 10^3^ cells/well) were seeded in 6-well culture plates and treated with violacein. After 12 days of incubation, the colonies that had formed were fixed with 3.7% formaldehyde by incubating for 20 min, and stained with 0.5% crystal violet reagent by incubating for 15 min. The stained colonies were washed with phosphate-buffered saline (PBS), and the number of visible colonies in each well was counted.

### 4.5. Nuclear Fluorescent Staining with DAPI

Huh7 cells (7 × 10^4^ cells/well) were seeded in 24-well culture plates and treated with violacein for 24 h. The cells were fixed with 3.7% formaldehyde by incubating for 15 min and stained with 5 μg/mL DAPI by incubating for 15 min. The fluorescent images were obtained using a fluorescence microscope (Optinity KI-2000F, Korea Lab Tech, Seong Nam, Korea).

### 4.6. Measurement of MMP

Huh7 cells (5 × 10^4^ cells/well) were seeded in 96-black-well culture plates and treated with violacein for 24 h. The cells were subsequently treated with 10 µg/mL of the JC-1 fluorescent dye and were incubated for 20 min. The fluorescence intensity of the J-aggregates that formed and the JC-1 monomers was detected at wavelengths of 530/590 nm (excitation/emission) and 485/528 nm, respectively, using a multimode microplate reader (BioTek, Winooski, VT, USA). Mitochondrial depolarization was indicated by a decrease in the ratio of intensity of red/green fluorescence.

### 4.7. Measurement of ROS

Huh7 cells (5 × 10^4^ cells/well) were seeded in a 24-well culture plate and treated with violacein for 6 h. The cells were stained with 15 µM DCFH-DA by incubating for 10 min. The fluorescent images were obtained using a fluorescence microscope (Optinity KI-2000F, Korea Lab Tech, Seong Nam, Korea), and the fluorescence density was analyzed using ImageJ software, version 1.5 (NIH).

### 4.8. Analysis of Apoptosis

Huh7 cells (2 × 10^5^ cells/well) were seeded in a 60 mm cell culture dish and treated with violacein for 24 h. The cells were harvested, washed with PBS, and stained with 100 µL of Muse^®^ Annexin V & Dead Cell reagent, according to the manufacturer’s instructions. The stained cells were analyzed using a Guava^®^ Muse^®^ Cell Analyzer (MuseSoft_V1.8.0.3; Luminex Corporation, Austin, TX, USA).

### 4.9. Cell Cycle Analysis

Huh7 cells (1 × 10^5^ cells/well) were seeded in a 60 mm cell culture dish and treated with violacein for 24 h. The cells were harvested, washed with PBS, and fixed with 70% ethanol at −20 °C for 3 h. After washing with PBS, the cells were stained with 200 µL of Muse^®^ Cell Cycle reagent, according to the manufacturer’s instructions. The stained cells were analyzed using a Guava^®^ Muse^®^ Cell Analyzer.

### 4.10. Western Blot Analysis

The cells were lysed using RIPA buffer, supplemented with protease and phosphatase inhibitors (ATTO, Tokyo, Japan). The concentrations of the proteins were determined using the Pierce^®^ BCA Protein Assay Kit (Thermo Fisher Scientific, Inc., Rockford, IL, USA). Equal amounts of cell lysates were separated by 7.5–15% sodium dodecyl sulfate-polyacrylamide gel electrophoresis (SDS-PAGE) and subsequently transferred to polyvinylidene difluoride (PVDF) membranes (EMD Millipore, Hayward, CA, USA) using standard electroblotting procedures. The blots were blocked with 5% skim milk in Tris-buffered saline with Tween-20 (TBST) at room temperature for 1 h, and immunolabeled with the primary antibodies against cleaved capase-9, cleaved caspase-3, PARP, p21, p53, phospho-ERK1/2, ERK1/2, phospho-AKT, AKT, phospho-STAT3, STAT3, ALDH1A1, integrin α6, Sox2, Oct4, Nanog, CD133, α-tubulin (dilution 1:2000), and β-actin (dilution 1:10000) by incubating overnight at 4 °C. After washing thrice with TBST, the membranes were incubated with horseradish peroxidase-conjugated anti-rabbit or anti-mouse (dilution 1:3000) secondary antibody for 1 h at room temperature. Immunolabeling was detected using an enhanced chemiluminescence (ECL) kit (Bio-Rad Laboratories, Hercules, CA, USA), according to the manufacturer’s instructions. The density of the bands was analyzed using ImageJ software, version 1.5 (NIH).

### 4.11. Tumorsphere Forming Assay

Huh7 and Hep3B cancer stem-like cells (3 × 10^3^ cells/well) were seeded in 96-well culture plates using serum-free media with EGF and bFGF, and they were treated with violacein at various concentrations. After 7 days of incubation, the size and number of tumorspheres were determined using an optical microscope (Olympus, Tokyo, Japan).

### 4.12. Statistical Analyses

The results are presented as the mean ± standard deviation (SD) of data obtained from at least three independent experiments. The differences among the groups were analyzed by analysis of variance (ANOVA), calculated using the SPSS software, version 9.0 (SPSS Inc., Chicago, Ill., USA). Post hoc analysis was performed using Tukey’s test. Statistical significance was considered at *p* < 0.05.

## 5. Conclusions

This study is the first to demonstrate the anticancer effect of violacein against HCC cells and elucidate the molecular mechanism underlying its antitumor activity. The results demonstrate that violacein effectively inhibited the proliferation of HCC cells by inducing cell cycle arrest at the sub-G1 phase and triggering apoptosis. The apoptosis induced by violacein was associated with nuclear condensation; loss of MMP; increased generation of ROS; activation of caspase-9, caspase-3, and PARP; upregulation of p53 and p21; and downregulation of AKT and ERK1/2 signaling. Furthermore, violacein significantly suppressed the proliferation and formation of tumorspheres of HCC stem-like cells by reducing the expression of HCC stemness markers, including CD133, Sox2, Oct4, and Nanog, and by inhibiting the STAT3/AKT/ERK signaling pathways. In conclusion, these findings suggest that violacein has the chemotherapeutic potential to effectively suppress HCC by targeting both the proliferation and stemness of HCC cells.

## Data Availability

The data that support the findings of this study are available from the corresponding author upon reasonable request.

## Supplementary Materials

The following are available online at https://www.mdpi.com/article/10.3390/ijms221910731/s1.
