# Supplementary material for: The Natural Pigment Violacein Potentially Suppresses the Proliferation and Stemness of Hepatocellular Carcinoma Cells In Vitro
Source: Int J Mol Sci. 2021 Oct 3;22(19):10731. doi: 10.3390/ijms221910731 (PMC8509727; doi:10.3390/ijms221910731)
Supplement: Supplementary file 1 [file ijms-22-10731-s001.zip › ijms-1332877-SI.pdf]

*Article*

# The Natural Pigment Violacein Potentially Suppresses the Proliferation and Stemness of Hepatocellular Carcinoma Cells In Vitro

**Yu Jin Kim [1,†], Nayeong Yuk [2,†], Hee Jeong Shin [1] and Hye Jin Jung [1,2,3*]**

[1] Department of Life Science and Biochemical Engineering, Sun Moon University, Asan 31460, Korea; petaldew17@naver.com (Y.J.K.); gmlwjd903@naver.com (H.J.S.)

[2] Department of Pharmaceutical Engineering and Biotechnology, Sun Moon University, Asan 31460, Korea; nayeong7249@naver.com (N.Y.)

[3] Genome-Based BioIT Convergence Institute, Asan 31460, Korea

* Correspondence: poka96@sunmoon.ac.kr (H.J.J.); Tel.: +82-41-530-2354; Fax: +82-41-530-2939

† These authors contributed equally to this work.

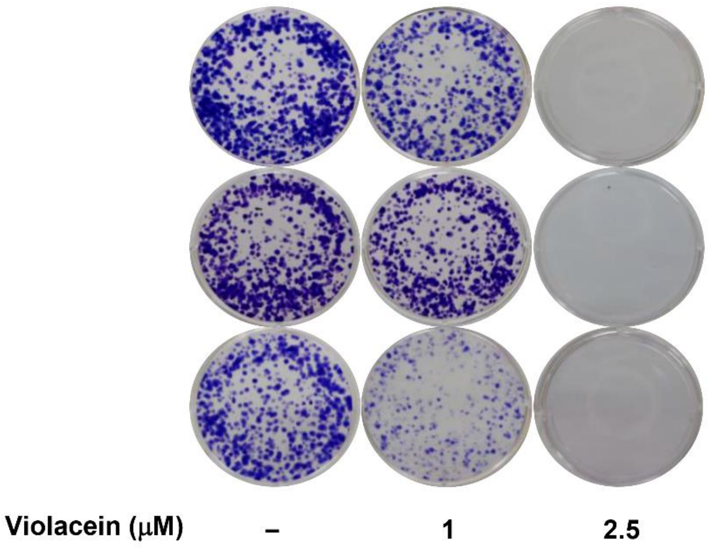


**Figure S1.** Violacein inhibits the colony forming ability of Huh7 HCC cells. The cells were incubated in the absence or presence of violacein (1 and 2.5 μM) for 12 days. The cell colonies were detected by crystal violet staining.

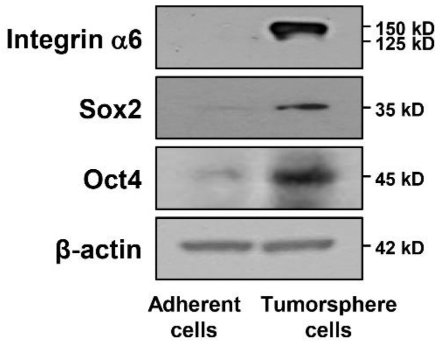


**Figure S2.** Expression levels of key stemness-related markers in Huh7 cells cultured under different conditions. The CSC marker expression was analyzed by Western blotting. β-actin was used as a loading control. **Left lane:** Huh7 adherent cells cultured in 10% FBS-supplemented media. **Right lane**: Huh7 tumorsphere cells cultured in serum-free media containing EGF and bFGF.

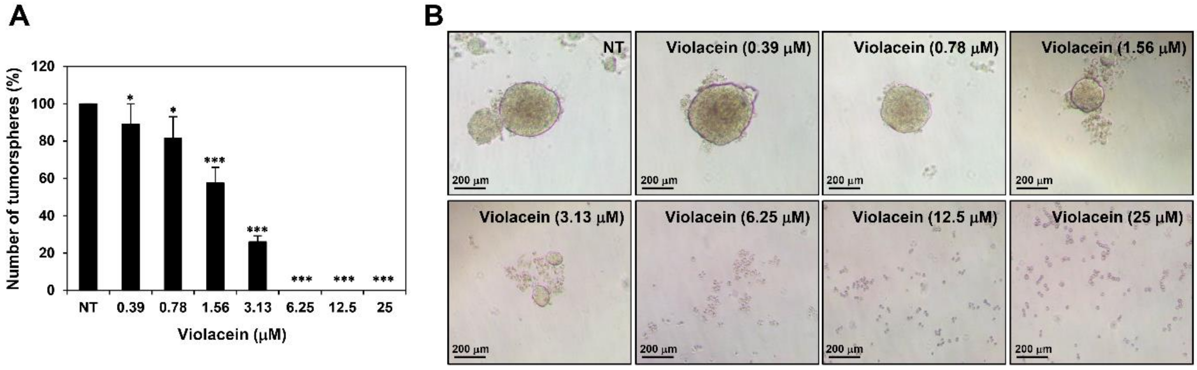


**Figure S3.** Violacein inhibits the formation of tumorspheres of Hep3B cancer stem-like cells. (**A,B**) Effect of violacein on the tumorsphere forming ability of Hep3B cancer stem-like cells. The cells were treated with violacein at various concentrations (0–25 μM) and incubated with the CSC culture media for 7 days. The number of tumorspheres in each well was counted under an optical microscope. * $p < 0.05$, *** $p < 0.001$ vs. the control.
